# Notched Sound Alleviates Tinnitus by Reorganization Emotional Center

**DOI:** 10.3389/fnhum.2021.762492

**Published:** 2022-01-28

**Authors:** Bixue Huang, Xianren Wang, Fanqing Wei, Qiyang Sun, Jincangjian Sun, Yue Liang, Huiting Chen, Huiwen Zhuang, Guanxia Xiong

**Affiliations:** ^1^Department of Otorhinolaryngology, The First Affiliated Hospital, Sun Yat-sen University, Guangzhou, China; ^2^Institute of Otorhinolaryngology, Sun Yat-sen University, Guangzhou, China

**Keywords:** tinnitus, fNIRS, sound therapy, neural plasticity, BA46

## Abstract

**Background:**

Tinnitus is a common disease, and sound therapy is an effective method to alleviate it. Previous studies have shown that notched sound not only changes levels of cortical blood oxygen, but affects blood oxygen in specific cerebral cortical areas, such as Brodmann area 46 (BA46), which is associated with emotion. Extensive evidence has confirmed that tinnitus is closely related to emotion. Whether notched sound plays a role in regulating the emotional center is still unclear.

**Methods:**

This study included 29 patients with newly diagnosed chronic tinnitus who were treated with notched sound. Functional near-infrared spectroscopy (fNIRS) was conducted before and after treatment to observe bilateral changes in cortical blood oxygen in the cerebral hemispheres. We compared the changes in connectivity between the two regions of interest (the superior temporal gyrus and BA46), as wells as other cortical regions before and after treatment.

**Results:**

The results showed (1) That global connectivity between the bilateral auditory cortex of the superior temporal sulcus and the ipsilateral cortex did not change significantly between baseline and the completion of treatment, and (2) That the connectivity between channel 14 and the right superior temporal sulcus decreased after treatment. The overall connectivity between the right BA46 region and the right cortex decreased after treatment, and decreases in connectivity after treatment were specifically found for channels 10 and 14 in the right parietal lobe and channels 16, 20, 21, and 22 in the frontal lobe, while there was no significant change on the left side. There were no significant changes in the questionnaire measures of tinnitus, anxiety, or depression before and after treatment.

**Conclusion:**

The results of the study indicate that cerebral cortex reorganization occurs in tinnitus patients after submitted to treatment with notched sound for 1 month, and that notched sound decreases the connectivity between the auditory cortex and specific brain regions.

**Significance:**

Notched sound not only regulates the auditory center through lateral inhibition, but also alleviates tinnitus by reorganizing the emotional control center.

## Highlights

- Functional near-infrared spectroscopy was used to observe the cortical function of tinnitus patients.
- Several non-auditory cortices showed stable reorganization after the sound therapy.
- Notched sound alleviated tinnitus by reorganizing the emotion-related cortical center.

## Introduction

Tinnitus, which is a common disease that afflicts people, is defined as subjective sound perception in the absence of an external sound source (Bauer and Solomon, 2018). Patients who suffer from long time tinnitus also often suffer from some negative effects (Bhatt et al., 2017) include insomnia, anxiety, depression, and even suicidal tendencies. In addition, tinnitus imposes a significant financial burden on the medical system and has a significant economic impact on society (Wu et al., 2016).

There are many methods to treat tinnitus, including drug therapy, cognitive behavioral therapy (CBT), masking sound therapy, transcranial magnetic stimulation (TMS), vagus nerve stimulation therapy (VNS), and Chinese acupuncture. However, few methods have been proven to be effective by evidence-based medicine. Sound therapy is a non-invasive method that is worth trying and has been recommended by the 2014 American Academy of Otolaryngology–Head and Neck Surgery Foundation (AAO-HNSF) clinical practice guidelines for tinnitus (Tunkel et al., 2014). Sound therapy involves the use of a specific masking noise to reduce the contrast between tinnitus signals and background activity in the auditory system, thereby reducing the patient’s perception of the tinnitus signals (Hobson et al., 2012).

There are many choices of sound to use, such as broadband noise (e.g., white noise and pink noise), nature sounds, and sounds adjusted to tinnitus frequency, such as narrow-band noise or notched sound. Different masking sounds have different spectral characteristics and their therapeutic mechanisms vary. Notched sound is a customized sound that removes the frequency near the tinnitus frequency and replaces it with white noise (Stein et al., 2016).

Notched sound was first proposed by Pantev et al. (2012), when using a magnetoencephalography (MEG) to study the influence of such sound on the cerebral cortex. The study found that notched sound could activate neighboring neurons and reduce the activity of the cerebral cortex in relation to the frequency of the sound, where the gap is created by lateral inhibition. Many studies on the practical application of notched sound to treat patients with tinnitus have found that customized notched sound treatment can significantly reduce the perceived loudness of tinnitus and cause changes in the excitability of the auditory cortex (Okamoto et al., 2009; Stein et al., 2016).

The evaluation of the brain function of tinnitus patients has become a research hotspot, worldwide, in order to understand the mechanism of tinnitus better (Eggermont and Roberts, 2004; Eggermont, 2007, 2015a,b; Schlee et al., 2009; Moazami-Goudarzi et al., 2010; Vanneste and De Ridder, 2012). Common techniques used to study the functional state of the brain include functional magnetic resonance imaging (fMRI), electroencephalography (EEG), magnetoencephalography (MEG), and molecular imaging technology-positron emission tomography (PET/CT). Functional brain imaging techniques, such as fMRI and EEG, provide a theoretical basis for understanding the cognitive functions of the brain. Previous studies have shown that tinnitus is associated with neural changes in central auditory pathways associated with non-auditory brain regions. Norena and Eggermont (2005) found that the resting state network in the hypothalamus and Heschl’s gyrus was significantly enhanced in tinnitus patients when compared to healthy controls, and was correlated with reported loudness of tinnitus. In addition, it was demonstrated after regular masking therapy, the cortical activity associated with tinnitus decreased significantly. Song et al. (2012) and Schecklmann et al. (2013, 2014), who used PET to study the metabolism of the cortex in the resting state of tinnitus patients, found that the metabolism of the primary auditory cortex was increased in tinnitus patients. Research on the brain function in patients with tinnitus has found that tinnitus are associated with changes in many areas of the cortex, including the ventromedial prefrontal cortex, left inferior prefrontal cortex, insula, hypothalamus, as well as other areas. Issa et al. (2016) found that the control group showed inactivity in both auditory and non-auditory regions during the silent phase between two sound stimulus phases, while tinnitus patients showed activity. Ahveninen et al. (2017) found that after submitted to sound stimulation, the resting-state functional connections between the auditory and non-auditory cortices increased in patients with tinnitus and decreased in healthy controls.

We used functional Near-Infrared Spectroscopy (fNIRS) to study changes in the auditory cortex of patients when they were temporarily stimulated by three different types of masking noise, namely, narrow band noise, white noise, and notched sound (Sun et al., 2020). The results showed that a masking sound could cause changes in blood oxygen amplitude in the corresponding auditory region of interest in the cortex. The three different masking sounds had different cortical activity characteristics, suggesting that different sounds may have different mechanisms of therapeutic effect. Notched sound can cause changes in several non-auditory cortical regions, aside from the auditory center, including the inferior frontal gyrus. This means that notched sound may not only alleviate tinnitus through lateral inhibition of the auditory center, but also cause changes in the reticular structure of other areas of the non-auditory cortex during treatment.

There were study found that (Song et al., 2012) the excitability of the inferior frontal gyrus of patients with tinnitus was generally greater than that of healthy people. This region corresponds to Brodmann area 46, which is a sub-region located in the inferior frontal gyrus of the cerebral cortex. Previous studies of this region indicate that it is closely related to emotional processing and emotion regulation (Phan et al., 2005; Goldstein et al., 2007), and that it is actively involved in the suppression of negative emotions. That is to say, when individuals actively suppress negative emotions, this leads to increased cortex activity in this area. Changes in this area have been seen in people with obsessive-compulsive disorder, depression (Gilbert et al., 2008), and schizophrenia (Webler et al., 2020). Since much evidence has shown that tinnitus is strongly related to emotion, changes in the affect-related cortex may be closely related to the occurrence and development of tinnitus, and its mechanism may involve an interaction between the auditory pathway and the emotion center through the network structure.

Therefore, we used fNIRS to explore whether notched sound could regulate tinnitus by affecting the emotional center.

## Materials and Methods

### Participants

Patients who reported suffering from subjective tinnitus for more than 3 months were selected from the outpatient Department of Otorhinolaryngology of The First Affiliated Hospital of Sun Yat-sen University. The outpatient doctors assessed each patient for tinnitus. Collection of some essential information was also conducted at this time, such as a routine medical history, examination of the ear, including a physical examination of the external auditory canal and tympanic membrane. In addition, audiology tests were conducted, including conventional pure-tone audiometry (PTA) (125 Hz–8 kHz) (Vielsmeier et al., 2015), immittance measurement, otoacoustic emission. CT/MRI imaging examinations were also performed according to the specific condition. After completing these procedures, the following inclusion and exclusion criteria were used to select participants.

The inclusion criteria were: (1) Age ≥18 years old; (2) Normal hearing or mild hearing loss (≤25 dB) in both ears, based on the average of pure tone audiogram results at 500, 1,000, 2,000, and 4,000 Hz; and (3) The main tinnitus frequency was between 4,000 and 8,000 Hz. The exclusion criteria were: (1) Patient who had pulsatile tinnitus, Meniere’s disease, otosclerosis or a history of middle ear surgery; (2) Patient who had hearing hypersensitivity or were unable to complete routine audiology examinations and tinnitus detection; (3) Patients who suffered from severe mental disorders and were unable to cooperate with the Pure-tone Average (PTA) threshold test and tinnitus detection; (4) Patients who had medical and neurological related diseases, epilepsy, or a history of chronic headaches; (5) Patients who had a history of alcohol or drug abuse; (6) Patients who had a history of head or neck trauma, such as temporal bone fracture; and (7) Patients with poor compliance and difficulty during follow-up.

A total of 29 tinnitus patients were recruited for this study (Table 1). Among them, were 18 males and 11 females, age 19–52 years, with an average age of 31.45 ± 9.68 years. The course of disease ranged from 3 to 36 months, with an average course of 10.90 ± 9.34 months. All subjects gave written informed consent.

**TABLE 1:** Clinical data.

Number	Gender	Age (years old)	Tinnitus pitch (Hz)	Side	Duration (months)
1	Male	24	6000	Left	4
2	Male	40	6000	Left	6
3	Male	37	6000	Right	6
4	Male	35	6000	Both	5
5	Female	40	6000	Both	12
6	Female	20	4000	Left	36
7	Female	25	6000	Both	4
8	Female	27	4000	Right	6
9	Male	27	8000	Both	6
10	Male	19	6000	Both	3
11	Male	29	8000	Both	12
12	Male	31	4500	Left	3
13	Male	44	6000	Both	5
14	Male	21	5500	Right	8
15	Male	26	6000	Left	5
16	Female	38	6750	Right	24
17	Male	22	6000	Left	12
18	Female	32	5500	Left	7
19	Male	21	8000	Right	12
20	Male	29	8000	Both	24
21	Female	19	6000	Left	6
22	Female	36	8000	Both	12
23	Male	52	8000	Both	4
24	Female	22	6000	Both	4
25	Male	25	6000	Both	6
26	Female	48	6000	Left	6
27	Male	32	8000	Left	18
28	Male	51	5500	Both	36
29	Female	40	8000	Right	24

### Audiometric Evaluations and Tinnitus Tests

The PTA threshold test and tinnitus pitch matching examination of all patients were performed in a soundproof room, and the examination environment met the national acoustic standards. The PTA threshold test used an audiometer (Natus Hearing & Balance, Copenhagen, Denmark) to measure the patient’s hearing threshold in the frequency range of 250–8,000 Hz. TinniFit (Bozy Medical Technology Limited, Foshan, China) was used to present pure tones of different frequencies to patients through headphones, and patients were asked to determine which tone was most similar to their tinnitus, to determine their tinnitus pitch.

### Questionnaires

All the patients were asked to complete a series of tinnitus-related questionnaires and psychosocial questionnaires during the last week before sound therapy (i.e., the baseline measures) and after sound therapy. The effect of tinnitus on subjects was assessed with the Tinnitus Handicap Inventory (THI). THI include three subscales of emotional, functional and catastrophe part. In addition, patients were asked to complete the Beck Anxiety Inventory (BAI), which evaluated their recent level of anxiety, and the Center for Epidemiologic Studies-Depression (CES-D) Scale, which evaluates severity of depression.

### Testing Environment and Optode Localization

**FIGURE 1:**
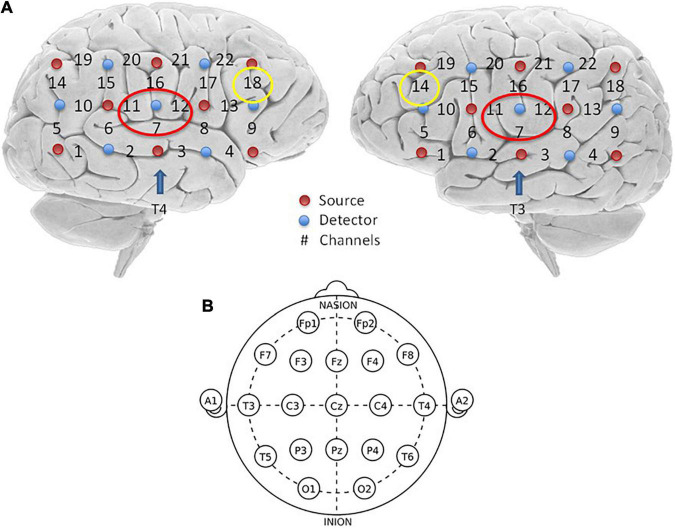
Optode localization and the channel of ROI. **(A)** Red dots on both hemispheres represent source emitters, blue dots represent detectors, and the numbers represent the channels. There were eight emitters, seven detectors, and 22 channels on each hemisphere. Channels marked with red circles are bilateral channels 7, 11, and 12, which are the interest regions of the superior temporal sulcus selected in this study. Channels marked with yellow circles are channel 18 on the left and channel 14 on the right, which are the interest regions of BA46 selected in this study. **(B)** Two arrays of probes were placed in the left and right temporal regions, respectively, and placed on each subject’s head, according to the relevant standard position of the International 10–20 System (Obrig and Villringer, 2016). The upper channel was placed horizontally at T3/T4 and vertically at C3/C4.

Patients were tested in a soundproof environment. The data were obtained using a Hitachi ETG4000 (Hitachi Medical Corporation, Tokyo, Japan) system, which had 30 probes (16 light sources and 14 detectors) arranged in two configured 3 × 5 arrays. The light source and detector were spaced at a fixed distance of 3 cm. Two arrays of probes were placed in the left and right temporal regions of each patient’s head, according to the relevant standard position of the 10–20 system (Obrig and Villringer, 2016). The upper channel was placed horizontally at T3/T4 and vertically at C3/C4. Infrared light was produced at two wavelengths (695 nm and 830 nm) and collected the hemoglobin-dependent signals at a sampling rate of 10 Hz (Figure 1).

All the probes were carefully checked before the test, to insure they had good contact. The hair between the probe and scalp was moved before the test in order to make the probe directly contact the scalp to obtain better signals. Patients were told not to touch the probe during the test, in order to avoid head and body movements that could cause artifacts due to probe displacement.

### Definition of Region of Interest

We selected two regions of interest (ROI) for our research based on previous studies on audiology and the resting state of the cerebral cortex. The areas of interest included the bilateral superior temporal sulcus (STC) and the bilateral Brodmann 46 area (dorsolateral prefrontal lobe) (Table 2). The bilateral superior temporal sulcus is one of the most common areas of interest to investigate auditory tasks, including channels 7, 11, and 12, on the left side and 7, 11, and 12 on the right side. According to previous studies, BA46 is an important cortical region involved in working memory, attention maintenance, and emotional processing (Aron et al., 2014). It includes channel 18 of the left cortex and channel 14 of the right cortex.

**TABLE 2:** Corresponding Brodmann areas and cortical regions for each channel.

Channel number		Brodmann areas	Anatomic location	Percent channels
Left	Right			
1	4	38	Frontal	18.2%
14	18	46	Frontal	
15	17	6, 9	Frontal	
19	22	8, 9	Frontal	
5	9	47	Frontal-temporal	13.6%
6	8	22, 44, 38	Frontal-temporal	
10	13	44, 45	Frontal-temporal	
2	3	21	Temporal	13.6%
3	2	21, 22	Temporal	
8	6	21, 22, 37	Temporal	
11	12	1, 2, 3, 4, 6, 43	Frontal-parietal	18.2%
16	16	1, 2, 3, 4, 40	Frontal-parietal	
20	21	4, 6	Frontal-parietal	
21	20	1, 2, 3, 7, 40	Frontal-parietal	
7	7	22, 42	Temporal-parietal	22.7%
12	11	40, 41, 42	Temporal-parietal	
13	10	39, 40	Temporal-parietal	
17	15	39, 40	Temporal-parietal	
22	19	7, 39, 40	Temporal-parietal	
18	14	19, 39	Occipital-temporal	4.6%
4	1	19, 37	Occipital	9.1%
9	5	19, 18	Occipital	

### Sound Therapy

After the patients provided the preliminary clinical data, they were asked to listen to the sound that was un-interrupted for 30 min once a day for 30 days. Then, they returned to the clinic on day 31 for a return visit.

**FIGURE 2:**
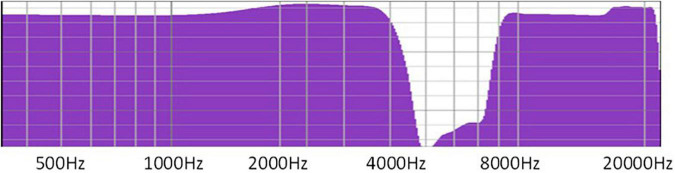
Notched sound was created by band-stopping the 4,000–8,000 Hz spectrum using white noise.

Notched sound was selected as the sound source of the patient’s therapy. Notched sound was created by band-stopping the 4,000–8,000 Hz spectrum based on white noise (Figure 2). Studies have shown that this kind of sound source can be used for masking-sound therapy, through lateral inhibition to reduce the over-activity of the nerve, and to reduce tinnitus-related cortical activity and tinnitus loudness (Haab et al., 2019), by achieving cortical reorganization.

### Data Preprocessing and Statistical Analyses

Resting fNIRS data were collected twice before the sound therapy (baseline) and again during the return visit 1 month after the beginning of therapy. After the probes were fixed, patients were instructed to remain stationary and to avoid limb movements. Then, a 10-min recording was presented.

All the data preprocessing was done through the software package for fNIRS-based human functional connectome studies called “functional connectivity analysis tool for near-infrared spectroscopy data” (FC-NIRS; Xu et al., 2015) of MATLAB software (MathWorks, MA, United States). The original data were converted to concentration changes using the modified Beer–Lambert law (MBLL). Oxygenated hemoglobin (HBO) and deoxyhemoglobin (HBR) were measured in each group.

First, we converted the .csv file to the .nirs format. Then, steps were conducted to process the .nirs file as a MATLAB file .proc format. The data packet of the file contained the original data of the .nirs file, changes in optical density, and data from the detection channel. Changes in HBO, HBR, and total hemoglobin (HBT) over time were also measured. After removing the influence of motion artifacts through quality control, the final connectivity was calculated.

We used the pre-selected regions of interest as seeds in the calculation of the connectivity between channels, used the seed-based correlation method to estimate the strength of the paired relationships between the seed regions and all other regions in the brain, and performed Pearson correlations on them. After the data for each subject were obtained, SPSS software (version 20.0; SPSS Inc., Chicago, IL, United States) was used to perform a paired *t*-test on the connectivity data between the ROI and each channel before and after treatment (i.e., pre-/post-treatment).

In addition, *t*-tests were performed on the scale data of the subjects pre- and post-treatment. A *P* < 0.05 was considered statistically significant for all the analyses.

## Results

### Clinical Data

**FIGURE 3:**
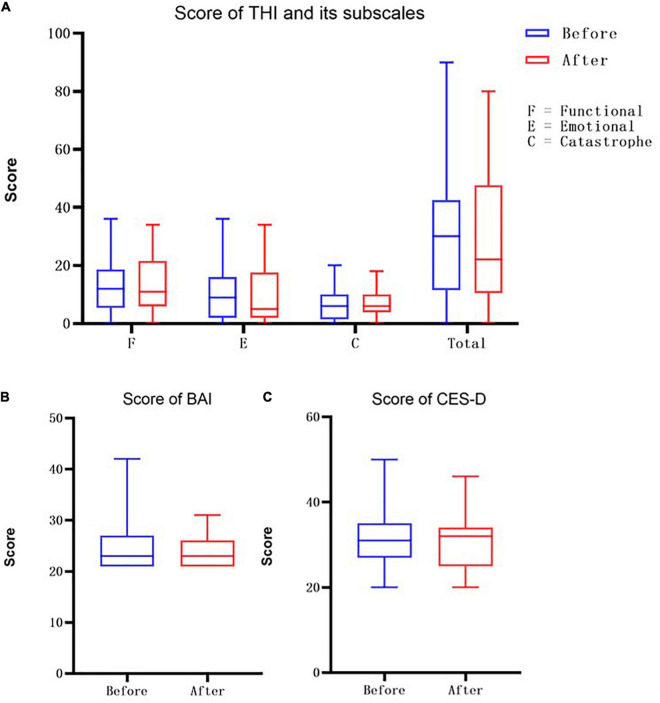
**(A)** Here is the score of THI scale, where F represents the functional score, E represents the emotional score, and C represents the score of catastrophe, the last one represents the Total THI score. The blue bars are the socres before the treatment and the red bars are the socres after receving the sound therapy. **(B)** The score of Beck Anxiety Inventory (BAI). **(C)** The score of Center for Epidemiologic Studies-Depression (CES-D) Scale.

The analyses of the questionnaires found that there were no significant differences in the THI, BAI, or CES-D before and after sound treatment. Also, there were no significant differences in the THI subscales, such as the emotional, functional and catastrophe pre-/post-treatment, and the *P* values were not significant. However, as can be seen in Figure 3, the medians of the total score and the emotional subscale score showed a downward trend.

### Connectivity Between Region of Interests and Other Areas

We analyzed the connectivity between the two ROIs and the channels on the ipsilateral cortex. Since the STC on both sides contain channels 7, 11, and 12, STC was used as a single summary of this region in the table. The BA46 region has different channels on both sides, with channel 18 on the right side and channel 14 on the left side, so it is marked separately, according to the channels.

A *t*-test was performed on the values of the different ROIs and the connectivity of all other channels on the ipsilateral cortex, which were divided into two groups before and after treatment. The average connectivity values of all the patients in the same channel at different stages were recorded and listed in the table.

**FIGURE 4:**
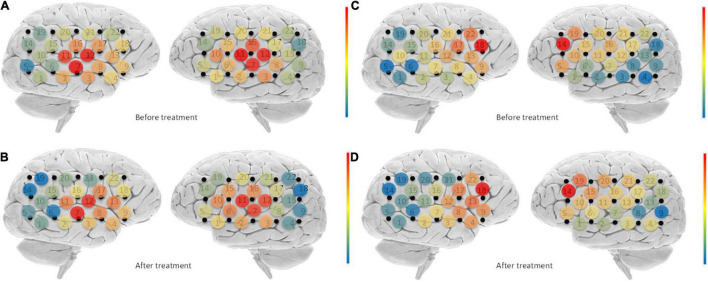
Changes in connectivity before and after treatment. Blue indicates low channel connectivity to the ROI in this area, and red indicates higher connectivity. Comparison of STC before and after treatment **(A,B)**, found that the connectivity of right channel 14 was significantly reduced after treatment. For BA46 **(C,D)**, the connectivity of channels 10, 14, 16, 20, 21, and 22 on the right side was significantly reduced after treatment.

No significant differences were found in the overall connectivity of each lateral STC interest region before and after treatment in the pooled analysis of the connectivity between each unilateral STC interest region and each channel in the respective hemispheric cortex (Table 3) in the overall connectivity of each lateral STC interest region before and after treatment. However, there was a consistent downward trend on both the left and right sides. Analyses of STC and related connectivity of specific channels (Table 3 and Figure 4) showed there was no significant changes in the connectivity with all other channels before and after treatment in the left hemisphere. Yet, the connectivity in the right hemisphere of this region and channel 14 decreased significantly after acoustic therapy (*P* = 0.0452, <0.5). It can be seen in the corresponding table (Table 2) of the channel and Brodmann regions listed above that right channel 14 belongs to the BA19 and BA39 regions in the occipital-temporal region.

**TABLE 3:** Connectivity between STC and different channels.

Connectivity between different Connectivity between different channels of the right cerebral hemisphere				Connectivity between different channels of the left cerebral hemisphere			
	Before	After	*P*		Before	After	*P*
1-STC	0.3378	0.2616	0.3442	1-STC	0.5060	0.5245	0.8417
2-STC	0.5241	0.4049	0.3134	2-STC	0.5508	0.5986	0.5448
3-STC	0.5468	0.4845	0.5288	3-STC	0.6346	0.6019	0.7173
4-STC	0.4475	0.5017	0.5783	4-STC	0.4323	0.2912	0.1900
5-STC	0.1515	0.2132	0.5038	5-STC	0.4565	0.4675	0.9084
6-STC	0.2873	0.1100	0.0795	6-STC	0.5731	0.6676	0.2757
7-STC	0.8470	0.8220	0.6736	7-STC	0.8256	0.7821	0.4592
8-STC	0.5275	0.5866	0.4547	8-STC	0.5947	0.4341	0.0562
9-STC	0.4755	0.4865	0.9128	9-STC	0.4016	0.2425	0.1850
10-STC	0.3390	0.2721	0.5767	10-STC	0.6387	0.6413	0.9741
11-STC	0.7378	0.5981	0.1375	11-STC	0.8829	0.8268	0.1609
12-STC	0.8128	0.7700	0.3304	12-STC	0.8355	0.7713	0.1583
13-STC	0.5155	0.6057	0.3305	13-STC	0.4484	0.3214	0.2234
14-STC	0.2869	0.0935	**0.0452**	14-STC	0.3868	0.3935	0.9459
15-STC	0.3614	0.3309	0.7708	15-STC	0.5575	0.6177	0.5113
16-STC	0.5770	0.4197	0.0792	16-STC	0.7413	0.6394	0.0751
17-STC	0.5449	0.5866	0.6412	17-STC	0.4909	0.4658	0.7908
18-STC	0.4495	0.4285	0.8457	18-STC	0.2940	0.1435	0.0641
19-STC	0.2704	0.0873	0.0879	19-STC	0.4559	0.3897	0.4427
20-STC	0.3827	0.2951	0.2367	20-STC	0.5079	0.4935	0.8747
21-STC	0.3944	0.2615	0.2224	21-STC	0.5045	0.4412	0.4356
22-STC	0.3559	0.3901	0.7334	22-STC	0.4218	0.2633	0.1640
Total	0.4624	0.4096	0.3476	Total	0.5519	0.5008	0.3309

*The bold values indicates the degree of the connectivity between two areas, and with values approaching 1 indicating higher connectivity.*

The unilateral statistics for the BA46 region (Table 4) show that the overall connectivity between the right cortical and BA46 decreased significantly after treatment (*P* = 0.0301, <0.05), while there was no significant difference on the left side. The ROI did not change significantly on the left hemisphere before and after treatment. On the right side, channels 10 (*P* = 0.0148), 14 (*P* = 0.0089), 16 (*P* = 0.0170), 20 (*P* = 0.0247), 21 (*P* = 0.0011), and 22 (*P* = 0.0200) differed significantly before and after treatment, with all of the channels showing a decrease (Figure 4). Channel 10, which is located at the top of the temporal lobe, corresponds to BA39 and BA40. Channel 14, which is located in the occipital-temporal region, corresponds to BA19 and BA39. Of the six channels, channels 16, 20, and 21 are all located at the top of the forehead: channel 16 corresponds to BA1, BA2, BA3, BA4, and BA40; channel 20 corresponds to BA4 and BA6; and channel 21 corresponds to BA1, BA2, BA3, BA7, and BA40. Channel 22, which is located at the forehead, corresponds to BA8 and BA9.

**TABLE 4:** Connectivity between BA46 and different channels.

Connectivity between different channels of the right cerebral hemisphere				Connectivity between different channels of the left cerebral hemisphere			
	Before	After	*P*		Before	After	*P*
1-18	0.2207	0.1123	0.2399	1-14	0.2871	0.2803	0.9351
2-18	0.3351	0.2907	0.6725	2-14	0.2457	0.2755	0.7371
3-18	0.3230	0.3350	0.9125	3-14	0.2202	0.3207	0.1597
4-18	0.3737	0.4891	0.2913	4-14	0.1939	0.3241	0.1892
5-18	0.1526	0.1056	0.6196	5-14	0.5091	0.4276	0.4356
6-18	0.1597	0.0445	0.2937	6-14	0.2774	0.3317	0.4895
7-18	0.3824	0.2904	0.3991	7-14	0.3156	0.2825	0.7288
8-18	0.4151	0.5286	0.3156	8-14	0.2316	0.1837	0.5588
9-18	0.5131	0.5223	0.9189	9-14	0.2403	0.1286	0.2991
10-18	0.3789	0.1146	**0.0148**	10-14	0.4544	0.4126	0.6392
11-18	0.3450	0.1682	0.0912	11-14	0.3966	0.4072	0.9123
12-18	0.5232	0.4540	0.3808	12-14	0.3582	0.3806	0.8172
13-18	0.5180	0.6196	0.3625	13-14	0.2572	0.2543	0.9733
14-18	0.2698	0.0180	**0.0089**	14-14	1.0000	1.0000	–
15-18	0.3040	0.1811	0.1694	15-14	0.3788	0.5372	0.1007
16-18	0.4530	0.2250	**0.0170**	16-14	0.4571	0.3893	0.5203
17-18	0.6723	0.5761	0.2322	17-14	0.3357	0.3340	0.9840
18-18	1.0000	1.0000	–	18-14	0.2167	0.2808	0.3722
19-18	0.2077	0.0433	0.0868	19-14	0.5560	0.6543	0.2925
20-18	0.3395	0.0974	**0.0247**	20-14	0.3742	0.5033	0.1517
21-18	0.4108	0.1187	**0.0011**	21-14	0.3504	0.4124	0.5049
22-18	0.6617	0.4528	**0.0200**	22-14	0.3081	0.3107	0.9740
Total	0.4072	0.3085	**0.0301**	Total	0.3620	0.3832	0.6792

*The bold values indicates the degree of the connectivity between two areas, and with values approaching 1 indicating higher connectivity.*

## Discussion

In this study, the connectivity between cerebral cortical regions in tinnitus patients who received sound therapy (i.e., notched sound) changed after receiving continuous acoustic stimulation for 1 month. This change can be seen even in the resting state without sound stimulation, which suggests that the brain has been remodeled. Previous study showed that after receiving either pathological or physiological stimulation (Jain et al., 1998), the function of cerebral cortex of animals and humans will change not only excitability, but also persistently. And this kind of situation may be due to the changes in specific neurons and synapses. This reflects the ability of the body to learn and adapt to external stimuli, to some extent. The changes in resting cortical connectivity observed in the present study are consistent with this premise.

The use of notched sound was first proposed by Pantev et al. (2012) in 1999. After the subjects of this study listened to a notched sound continuously for 3 h every day for 3 days, MEG, which was used to study the neural functioning of the auditory cortex, found that the auditory induced magnetic field of specific brain channels decreased in individuals with tinnitus, compared with a control group. That study demonstrated that this kind of sound can reduce the cortical activity corresponding to the center-frequency of the notch, suggesting there was suppression of neural activity by lateral inhibition. Based on this theory, the researchers applied such a special sound to the treatment of tinnitus patients (Okamoto et al., 2009; Stein et al., 2015, 2016), and obtained a significant therapeutic effect, in which patients’ subjective perception of tinnitus loudness decreased. Some of these studies that further analyzed the functioning of the auditory cortex of patients, also found that the excitability of the auditory cortex decreased after long-term sound therapy. This finding was also confirmed in the present study. After treatment, the connectivity between the auditory cortex and the whole brain decreased, and there was a significant decrease in specific areas. The theoretical mechanism of this effect implies that tinnitus is caused by significant spontaneous discharges of the auditory cortex and its poor organization (Okamoto et al., 2009), which leads to the increased metabolism in the auditory cortex of patients with tinnitus (compared to normal people); the reduced excitability of the auditory cortex after treatment suggests that the cortex changes toward normalization.

Tinnitus is a disease that is closely related to emotion and psychological state. Therefore, clinicians usually observe that patients with tinnitus have an obvious tendency for anxiety that may be accompanied by fear, fatigue, sleep disorders, and many other accompanying symptoms, which can affect their mental health and quality of life. Although there is no drug available to reduce the loudness of tinnitus, clinicians often prescribe medications to patients that affect their psychological and physical symptoms, such as sleeping pills, anti-anxiety drugs, and antidepressants. Although none of these drugs is a routinely recommended treatment, their targeted use does reduce patients’ symptoms to some extent, by decreasing the negative emotions associated with tinnitus. In addition, the clinical practice guidelines for patients with annoying tinnitus that affects their work life, including the tinnitus guidelines of the American Academy of Otolaryngology-Head and Neck Surgery Foundation (AAO-HNSF; Tunkel et al., 2014), clearly recommend adaptation therapy for patients, as well as counseling and guidance as very important parts of treating tinnitus. Doctors give psychological guidance in clinical work; for example, correcting patients’ incorrect ideas about tinnitus, in order to reduce or eliminate patients’ anxiety and advising patients about ways to live with tinnitus, which can reduce the impact of tinnitus on their lives and improve their tinnitus to a certain extent.

The cerebral cortex showed higher activity in areas associated with emotion, such as the BA46. This higher cortical excitability represents the patient’s active participation in suppressing negative emotions. In other words, when individuals actively suppress negative emotions, this leads to increased cortex activity in this area (Gilbert et al., 2008; Webler et al., 2020). Thus, if we can reduce the cortical excitability of this area, this may indicate that patients have less exposure to negative emotions. For this reason, we chose the BA46 region as one ROI in the study, and we found that BA46 activity did change, showing a significant decrease.

Additionally, as many studies have shown that notched sound can improve symptoms of tinnitus, we hypothesize that notched Sound can affect the auditory cortex and the emotion-related cortical center (i.e., BA46), so as to reduce tinnitus. Based on this hypothesis about treatment effects on the emotional center involved in tinnitus, we asked whether the hypothesis can be extended and applied to clinical practice. For example, given the actual situation of patients, they should be given recommendations that can improve their emotional state, such as exercise to affect the emotional center, which is worthy of further study.

We chose to use fNIRS in this study to measure cortical changes because it utilizes non-ionizing radiation, and it has high temporal and spatial resolution. In addition, it is quiet and can be adapted to many forms of audiological tasks. Moreover, previous studies by our research group have demonstrated that it has sufficient sensitivity and specificity to detect cortical changes caused by different acoustic stimuli. Therefore, fNIRS is a powerful and advantageous detection tool for exploring induced cortical activity, which has provided a solid technical foundation for this study.

This study’s statistical analyses of scales for tinnitus patients found even though the THI showed a trend toward improvement, there were no significant pre-/post-treatment differences. This is somewhat inconsistent with the findings of previous studies that found perceived loudness and the THI of patients decreased significantly after receiving notched sound therapy (Okamoto et al., 2009; Stein et al., 2016). This might be because previous studies required patients to listen for as long as half a year, or even 1 year, whereas the sound therapy in this study only lasted for 1 month. In addition, studies on cortical reorganization indicate neural stimulation has a cumulative effect to some extent Pantev et al. (1999).

Therefore, although specific areas of reorganization were found in the cortex in the current study, the cumulative effect did not reach the level that can cause significant changes in subjective feelings. Subsequent studies should increase their sample size to make the differences more likely to be statistically significant, or extend the duration of treatment to increase the cumulative effects of stimulation in order to achieve better treatment effects.

In addition, as one of the most extensively validated questionnaires in the field of tinnitus research Newman et al. (1996), the THI is usually used to quantify the severity of tinnitus rather than measure the degree of distress associated with tinnitus, so the THI may not specifically reflect improvements in tinnitus-related emotions. Moreover, our analyses of anxiety scores revealed even though no obvious pre-/post-treatment difference was found, we observed that most of the patients had no obvious baseline symptoms, although they may have had mild anxiety or depression. That is to say, the impact of tinnitus on the subjects’ mood at baseline was not serious, even if the patients’ subjective experience of the perceived loudness of tinnitus and its influence on their life improved after treatment, the treatment would not necessarily cause significant changes on the scales.

There are also limitation based on our results. In this study, we only focus on notched sound, but lack of control group. In the future, we may further enroll tinnitus patients and healthy control and provide them with another kind masking noise, such as narrow-band noise, so as to further explore the mechanism of sound therapy.

## Conclusion

Notched sound treatment for 1 month can restructure the cerebral cortex of tinnitus patients. Notched sound can decrease connectivity between the auditory cortex and specific brain regions. Notched sound treatment not only regulates the auditory center through lateral inhibition, but it also improves tinnitus by reorganizing the emotional control center.

## Data Availability Statement

The raw data supporting the conclusions of this article will be made available by the authors, without undue reservation.

## Ethics Statement

The studies involving human participants were reviewed and approved by the Ethics Committee of The First Affiliated Hospital of Sun Yat-sen University. The patients/participants provided their written informed consent to participate in this study.

## Author Contributions

BH and XW contributed equally in the conception and design of the study, organized the database, and performed the statistical analysis. BH wrote the first draft of the manuscript. FW, QS, JS, YL, HC, and HZ wrote sections of the manuscript. All authors contributed to manuscript revision, read, and approved the submitted version.

## Conflict of Interest

The authors declare that the research was conducted in the absence of any commercial or financial relationships that could be construed as a potential conflict of interest.

## Publisher’s Note

All claims expressed in this article are solely those of the authors and do not necessarily represent those of their affiliated organizations, or those of the publisher, the editors and the reviewers. Any product that may be evaluated in this article, or claim that may be made by its manufacturer, is not guaranteed or endorsed by the publisher.
